# The Effectiveness of Antiplatelet Therapy and the Factors Influencing It in Patients with Acute Coronary Syndrome before and during the COVID-19 Pandemic

**DOI:** 10.3390/medicina59010084

**Published:** 2022-12-30

**Authors:** Ovidiu-Ionut Anchidin, Stefan Horia Rosianu, Ancuta Nemes, Mihai Aldica, Dan Blendea, Adrian Molnar, Horatiu Moldovan, Dana Pop

**Affiliations:** 1 Faculty of Medicine, Iuliu Hatieganu University of Medicine and Pharmacy, 400337 Cluj-Napoca, Romania; 2 “Niculae Stancioiu” Heart Institute, 400001 Cluj-Napoca, Romania; 3 Department of Cardiovascular Surgery, Bucharest Clinical Emergency Hospital, 014461 Bucharest, Romania; 4 Faculty of Medicine, Carol Davila University of Medicine and Pharmacy, 050474 Bucharest, Romania; 5 Department of Cardiology, Clinical Rehabilitation Hospital, 400437 Cluj-Napoca, Romania

**Keywords:** antiplatelet agents, acute coronary syndrome, platelet reactivity, LTPR, HTPR, COVID-19

## Abstract

*Background and Objectives:* Dual antiplatelet therapy (DAPT) is essential in the treatment of patients with acute coronary syndrome (ACS). The objective of this study was to evaluate the effectiveness of antiplatelet medication in our practice and to investigate the factors that influence it. *Materials and Methods:* A prospective cohort observational study was conducted, in which 193 patients with ACS were enrolled. The patients were stented in the catheterization laboratory between May 2019 and October 2020, before and during the COVID-19 pandemic, and were receiving DAPT. Their platelet functions were tested using a Multiplate Analyzer. In addition to this, clinical data, demographics, laboratory tests, and cardiovascular risk factors were also analyzed. *Results:* 43.46% of the patients treated with aspirin were found to be resistant to it. This phenomenon was more common in men (48.17% vs. 31.48%, *p* = 0.036), and it was associated with being under the age of 50 (OR: 2.08; 95% CI: 1.11–3.90) and weighing over 70 kg (OR: 3.00; 95% CI: 1.21–7.40). Most of the patients treated with clopidogrel were in the optimal treatment window, while about half of the patients treated with ticagrelor had an exaggerated pharmacological response. Among the laboratory parameters, leukocytosis and platelet count were found to be determinants of platelet reactivity for both the aspirin and ticagrelor treatments. *Conclusions:* Many patients treated with antiplatelet agents are outside of the treatment window. The results obtained showed that low doses of gastro-resistant aspirin tablets are ineffective, and their efficacy can be influenced by various clinical and laboratory factors. Patients receiving ticagrelor have significantly reduced platelet reactivity, influenced only by certain laboratory indicators. The pandemic significantly influenced the results of the platelet aggregation tests only in patients treated with clopidogrel.

## 1. Introduction

Cardiovascular diseases are the leading causes of morbidity and mortality worldwide, according to the World Health Organization [1]. Of these, acute coronary syndrome (ACS) is responsible for most deaths [2]. Cardiovascular diseases that develop in the course of atherosclerosis include not only ischemic heart disease (CHD) but also cerebrovascular disease and peripheral arterial disease [3]. The optimal treatment includes reperfusion therapy, mainly percutaneous coronary intervention (PCI), consisting of percutaneous balloon angioplasty with or without stent implantation, which has been established as the most efficient therapy for reducing major cardiac events (MACE) [3,4]. First, bare metal stents (BMS) were used, but they presented an increased risk of restenosis, especially in diabetic patients. The main mechanism of this is a proliferative process induced by local inflammation that consists of the proliferation, migration, and differentiation of the smooth muscle cells, resulting in neointimal hyperplasia [5]. This was largely counteracted by the introduction of first-generation drug-eluting stents (DES). However, these presented another impediment: intrastent thrombosis. However, with the introduction of a new generation of DES with an optimized design, intrastent thrombosis was significantly reduced according to the Swedish SCAAR registry [6]. Still, dual antiplatelet therapy (DAPT) plays a major role in preventing intrastent thrombosis and other secondary events [7]. Administering aspirin in low doses (75–100 mg) reduces major adverse cardiovascular events (MACE) in electively stented patients [8]. However, 15–30% of the patients treated with clopidogrel have an inadequate antiplatelet response detected on aggregation tests [9]. This translates into high on-treatment platelet reactivity (HTPR), which can be counteracted by the introduction of a new class of more potent P2Y12 inhibitors. These have been shown to be superior in reducing major ischemic cardiovascular events compared to clopidogrel in exchange for an increased rate of bleeding events [10,11]. Consequently, the latest European guidelines for the treatment of patients with ACS recommend administering ticagrelor or prasugrel in the first instance, and only in the case of serious side effects or the unavailability of the first two is the use of clopidogrel recommended [4,7].

In different situations, it is necessary to individualize the antiplatelet treatment according to the ischemic and hemorrhagic risk of the patient [12]. Different factors, such as demographic (age, race, and sex) and clinical factors (diagnosis at hospital admission; the time from the acute phase or from percutaneous coronary intervention (PCI)), associated diseases (diabetes, kidney failure, anemia, thrombocytopenia, and acute inflammatory or infectious states, including the one caused by the coronavirus (COVID-19)) and medications (anticoagulants; non-steroidal anti-inflammatory drugs (NSAIDs)) can influence platelet aggregation [12,13,14,15]. Based on these factors, different scores for estimating the ischemic and hemorrhagic risk were issued, and these can guide the initial or subsequent choice of treatment and its appropriate duration [16].

The most accurate means of evaluating the effectiveness of the treatment is to test the platelet function using different aggregometers [17,18]. While not recommended to be performed routinely, it can be useful in specific clinical situations, such as intrastent thrombosis or when reducing the risk of bleeding by switching to a weaker P2Y12 inhibitor (de-escalation regimen) is desired [12,16]. Based on the values offered by the aggregometry, the antiplatelet effect can be categorized into three categories: high on-treatment platelet reactivity (HTPR), which occurs in the case of ineffective antiplatelet treatment and leads to a high thrombotic and ischemic risk; low on-treatment platelet reactivity (LTPR), which occurs in the case of an exaggerated response to treatment and leads to a high risk of hemorrhage; and moderate on-treatment platelet reactivity, which reveals an optimal antiplatelet effect and is considered to be the “therapeutic window” [12]. To evaluate the effectiveness of antiplatelet medication in our practice and to identify the factors that influence it, we intended to conduct a study on stented patients with ACS. The first studies at the beginning of the COVID-19 pandemic showed that infection with SARS-CoV-2 was associated with thromboembolic complications. The risk of myocardial infarction was increased in the 7 days after COVID-19 diagnosis [19]. In one study, moderate-to-severe COVID-19 disease was associated with platelet hyperreactivity [20], while another study on critically ill patients with COVID-19 did not show an increased platelet aggregability despite the hypercoagulable state [21]. We intended to analyze the platelet aggregability in our patients with ACS in order to evaluate whether there was a change during the COVID-19 pandemic.

## 2. Materials and Methods

We conducted a prospective cohort observational study at the Cluj-Napoca Heart Institute, with the approval of the institution’s ethics committee, after verifying the study protocol. The patients were enrolled in the study between May 2019 and October 2020, after providing informed consent. During this period, the pandemic caused by the novel coronavirus (COVID-19) also appeared. The first cases in our country were registered at the beginning of 2020, and a complete lockdown began in March. We included patients with recent ACS, before and during the lockdown, who underwent emergency percutaneous revascularization with stent implantation. The patients were divided into two periods: before (T1) and during (T2) the COVID-19 pandemic lockdown. They were undergoing antiplatelet therapy using either aspirin and ticagrelor or clopidogrel. The choice of antiplatelet medication was at the discretion of the attending physician, following the medical practice guidelines. After the administration of the loading dose (300 mg aspirin, 180 mg ticagrelor, or 600 mg clopidogrel), the patients were administered either 75–100 mg of aspirin and 180 mg ticagrelor or 75 mg clopidogrel per day. At least 24 h after the initial loading dose, venous blood (1 mL) was collected, and the aggregation tests were performed following the new impedance method using a Multiplate Analyzer at the laboratory of the Heart Institute in Cluj-Napoca. The instrument detects changes in the electrical impedance of the whole blood, by analyzing the aggregation and adhesion of blood platelets on two surfaces with an integrated electrode located in the test cuvette. In the blood sample used for testing, hirudin was used as an anticoagulant, while adenosine diphosphate (ADP) and arachidonic acid (AA) were used as agonists. Two separate determinations were recorded, the average value of each being automatically calculated as the area under the impedance curve (AUC) in standardized units (U). We applied the technique as previously described [17,18]. The ADP platelet reactivity test values were categorized as follows: LTPR, which is less than or equal to 18 U; MTPR, which is between 19–45 U; and HTPR, which is greater than or equal to 46 U. The ASPI test was considered normal if the values were below 20 U. If it was equal to or greater than 20 U, it was considered HTPR, indicating resistance to the aspirin treatment, with a consecutive thrombotic risk [22,23].

In addition, the following clinical and paraclinical patient data were recorded: diagnosis and date of hospital admission, demographic data, cardiovascular risk factors (smoking, diabetes, hypertension, family history of cardiovascular disease, and dyslipidemia), and the results of blood tests that could influence the effectiveness of antiplatelet medications. In the first months of the lockdown, PCR tests were unavailable for diagnosing COVID-19 infection among the hospitalized patients with ACS in our service. Because of this, we cannot specify the number of infected patients. Nevertheless, we proposed an arbitrary analysis of the aggregometry values before and after the start of the pandemic in order to evaluate whether this had any generic influence on the aggregation state of patients with ACS.

The data were correlated with the obtained aggregation values (ASPI test and ADP test) to identify the factors influencing the inadequate response to antiplatelet therapy.

### Statistical Analysis

The possible factors influencing the response to antiplatelet medication were analyzed separately for ticagrelor and aspirin using univariate logistic regression. To qualify the association between these and the inadequate response observed, the odds ratio (OR) was reported, accompanied by the corresponding 95% confidence interval.

The normality of the data distribution was verified using the Kolmogorov–Smirnov test. The continuous variables were presented as mean ± standard deviation (SD) or median and interquartile range (IQR), according to the normality of the data. The categorical variables were represented in terms of frequency (number of cases) and percentages.

For the continuous variables, statistical comparisons were performed using the *t*-test or the Mann–Whitney U test, depending on the data distribution. In contrast, for the categorical variables, the chi-square test and Fisher’s exact test were used. For our study, we considered the results to be significant at a value of *p* < 0.05. All of the statistical calculations were performed using IBM SPSS version 26.

## 3. Results

In line with the criteria described above, 193 patients were registered for participation in the study. Of these, 57 (29.53%) were young, under the age of 50, and 138 (71.50%) were men. They mainly presented with ST-Segment Elevation Myocardial Infarction (STEMI) (65.80%). During the lockdown in Romania, 85 patients were enrolled in the study, representing 44.04% of the total number of patients registered for participation in the study (Table 1). Among the cardiovascular risk factors analyzed, the most common were dyslipidemia (70.81%), overweight/obesity (78.08%), hypertension (63.73%), and smoking (37.83%). Among the analyzed laboratory parameters, the serum creatinine value was excessive in 21.57% of patients, leukocytosis was present in approximately half of the patients, and 22.75% had platelet values above 291,000/µL (in the upper half of the normal range or above the upper limit) (Table 1).

### 3.1. Aspirin Treatment

**Figure 1:**
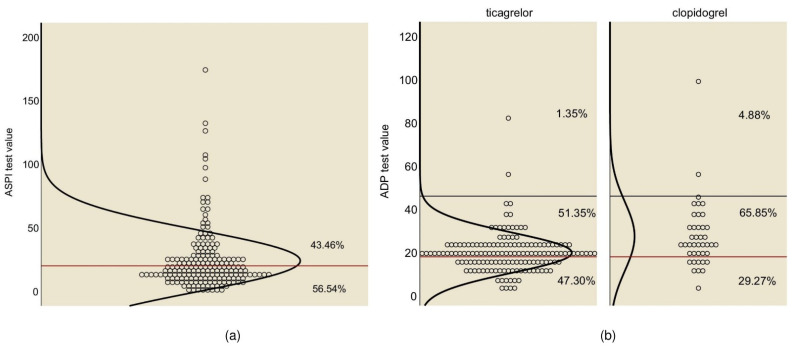
Response to antiplatelet therapy. (**a**) Response to aspirin treatment—the red line is drawn at 20, and the curve represents the normal distribution curve. (**b**) Response to P2Y12 inhibitor treatment, ticagrelor vs. clopidogrel—the red line is drawn at 18 and the black at 46, and the curve represents the distribution curve.

**Figure 2:**
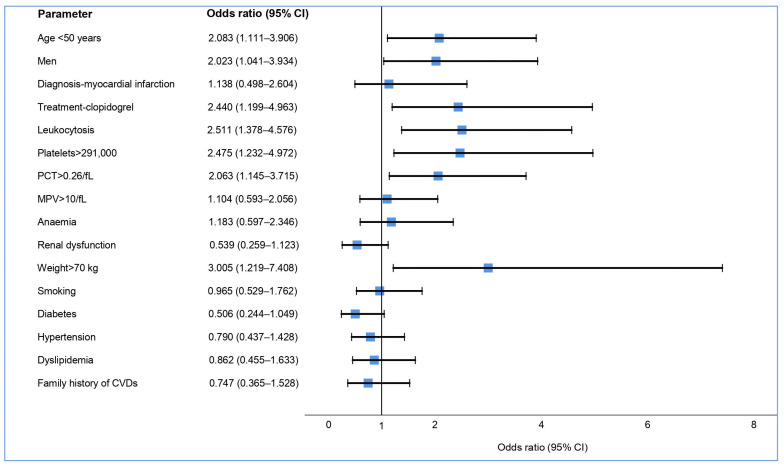
Risk factors for aspirin resistance.

Most patients (191, 98.96%) were treated with a modified-absorption form of aspirin at a dose of 75–100 mg/day. The AA platelet function test results indicated that 83 patients (43.46%) had a high ASPI test value (Figure 1a). The median ASPI test value was significantly higher in the patients administered with aspirin and clopidogrel than in those administered with aspirin and ticagrelor (Table 1). When compared to the aspirin-sensitive group, patients who were aspirin-resistant were significantly younger and more likely to be men (Table 2). Another risk factor in our study concerning aspirin resistance was weight over 70 kg (Figure 2).

Among the blood count parameters analyzed, it was observed that the presence of anemia did not influence the ASPI test values. However, leukocytosis led to significantly higher ASPI test values. Of the platelet indices analyzed, only the high platelet count and, implicitly, the plateletcrit were associated with increased aspirin resistance (Table 2, Figure 2). A slight increase in the percentage of patients resistant to aspirin was observed during the lockdown compared to the period prior to it, but did not reach the threshold of statistical significance (41.51% vs. 45.88%, *p* = 0.545) (Table 2).

### 3.2. Treatment with P2Y12 Inhibitors

Of the enrolled patients, 76.68% were treated with ticagrelor and 21.24% with clopidogrel. Ticagrelor was more often prescribed to young patients, patients with STEMI, and smokers (Table 1). The patients administered with clopidogrel more frequently experienced renal dysfunction to a certain degree (32.50% vs. 18.49%, *p* = 0.056). The general characteristics of the patients and the antiplatelet treatment followed are presented in detail in Table 1.

Regarding platelet activity, patients administered with clopidogrel had a median level ADP test value well above those administered with ticagrelor (25.00 vs. 19.00, *p* < 0.001) (Table 1). Only 2 of the 41 patients using clopidogrel (4.88%) and 2 of 148 patients using ticagrelor (1.35%) exhibited HTPR, as indicated by the ADP test, which did not allow for an adequate statistical analysis. After choosing the limit for the study, more patients treated with clopidogrel were inside the therapeutic window (MTPR) compared to those treated with ticagrelor (65.85% vs. 51.35%, *p* = 0.098), and much fewer fell into the category of LTPR (Figure 1b).

Among the clinical factors investigated, a statistically significant difference was only observed in terms of a patient’s age; the average age was higher in the group of patients at risk of bleeding (59.09 vs. 54.64, *p* = 0.02) (Table 3).

**Figure 3:**
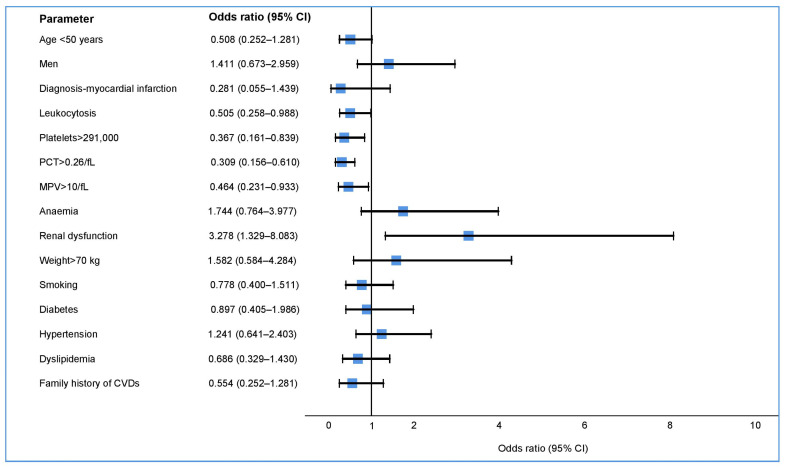
Risk factors for an elevated hemorrhagic risk in patients receiving ticagrelor.

Being below 50 years of age was a protective factor at the limit of significance for the occurrence of LTPR (*p* = 0.054) (Table 3, Figure 3). The latter was influenced in our study, especially by the laboratory parameters. Of these, the following platelet indices show statistical significance: platelet count, plateletcrit (PCT), mean platelet volume (MPV), leukocytosis, and renal dysfunction. A change in response to the treatment was also observed in patients undergoing treatment with ticagrelor, with a slight decrease in the percentage of patients with a high bleeding risk during the COVID-19 pandemic (50.00 vs. 44.29, *p* = 0.487) (Table 3). The median ADP test value was significantly higher in patients on clopidogrel during the COVID 19 pandemic than before the pandemic (22.00 vs. 31.00, *p* = 0.042) (Table 4).

## 4. Discussion

In daily clinical practice, aspirin resistance was more common than that described in controlled studies with a selected population, most likely due to the use of low doses and modified absorption formulas. This has been favored due to numerous clinical factors (men, young age, and overweight) as well as other factors, such as a high number of leukocytes and platelets. On the other hand, aggregation studies have revealed a much lower resistance to clopidogrel than that seen in other studies, with most patients being within the optimal antiplatelet treatment window. In patients treated with ticagrelor, it was found that almost half had an exaggerated antiplatelet response, exposing them to a significant risk of bleeding.

### 4.1. Aspirin Treatment

HTPR in patients treated with aspirin is a biomarker associated with poor prognosis due to the high risk of ischemic recurrences, especially due to increased mortality and intrastent thrombosis [24]. Aspirin is considered the cornerstone of ACS treatment. However, multiple studies have shown an extremely variable response, which is closely related to the method used, the selected population, and the time of blood collection. The literature lists more than 40 factors that may interact with the efficacy of aspirin, the most common of which are patient noncompliance, pharmacokinetics (underdose; limited absorption), and inefficient drug metabolism [25]. Most studies of this type have been carried out on patients on DAPT with clopidogrel, but the current standard treatment for stented patients with ACS implies a combination with more potent P2Y12 receptor inhibitors. In this context, two aspects need to be considered. First, the hemorrhagic risk for patients increases, and there is a tendency for clinicians to minimize the dose of aspirin to the limit of antiplatelet efficacy in order to counteract the risk. Next, there is evidence that the new antiplatelet agents interfere with the production of thromboxane A2 and with the action of aspirin [26]. In our study, almost 80% of the patients were treated with ticagrelor, and over 97% were treated with aspirin. Thus, we intended to evaluate, in addition to the effectiveness of the antiplatelet therapy in ACS patients stented in our hospital, certain factors that influence the response to these drugs based on aggregation analyses.

On examination of the ASPI test using arachidonic acid as an agonist, 43.46% of the patients had an inadequate response to aspirin, one of the highest rates reported in the literature. This may be primarily due to the dose administered, which was the minimum recommended by European guidelines (75 mg/day); however, the ACC/AHA guidelines recommend a dose of at least 82 mg/day to sufficiently reduce ischemic recurrence, compared to higher doses of up to 325 mg daily [27,28]. Moreover, in our cohort, gastro-resistant tablets have been administered in the case of both loading and maintenance doses, which have been shown to reduce intestinal absorption and to produce “pseudo-resistance” to aspirin [29,30].

Among the interaction factors analyzed, significant associations between age, gender, weight, and certain parameters of the blood count were observed (Table 2, Figure 2). Although there is an increase in platelet activity and a higher rate of ischemic events with age, and prothrombotic status is assigned to the elderly, a recent study showed that young people have higher ASPI test values, which was also confirmed in our study [13,31]. This may be due to some confounding factors, since the young patients in our study were predominantly overweight men who presented with STEMI. A meta-analysis published in 2018 of 10 randomized primary prevention trials showed that low doses of aspirin (75–100 mg) were only clinically effective in patients weighing less than 70 kg, and showed no benefit in the case of 80% of men and about 50% of women weighing over 70 kg [32]. Our results confirm the fact that increased body weight reduces the efficacy of aspirin. This can be explained by the increase in platelet activation and turnover, but also by the reduction in the bioavailability of aspirin due to the higher body mass distribution. The same mechanism of accelerating platelet turnover, as well as other mechanisms, such as inflammation, hyperglycemia, and insulin resistance, have been incriminated in diabetic patients exhibiting increased resistance to aspirin. Numerous studies have obtained contradictory results with regard to the link between diabetes and aspirin resistance [33,34]. One of the studies mentioned previously identified hypercholesterolemia as a predictor of aspirin resistance [34]. We found no association between aspirin resistance and diabetes or changes in the lipid profile.

An elevated leucocyte count has been associated in the case of patients with ACS with an increased ischemic risk. This can either be because it is a marker of a higher inflammatory status, a consequence of a larger infarction, or due to a prothrombotic status induced by the modulation of platelet activity, the formation of tissue factors, direct endothelial injury, or the activation of the extrinsic pathway [35,36]. In our cohort, leukocytosis was found in half of the patients, and was observed to be a risk factor for aspirin resistance (OR: 2.51; 95% CI: 1.37–4.57). In the specified pandemic context, we cannot exclude the association of leukocytosis with COVID-19 infection. The latter predisposes patients to thrombotic events due to excessive inflammation, endothelial activation and injury, platelet activation, and hypercoagulability [37]. The aggregation studies performed on COVID-19 patients have shown contradictory results. A study involving 41 patients who were not receiving antiaggregant treatment showed an increased aggregability in COVID-19 patients in response to different agonists [38]. Another study that used impedance-based aggregometry with Multiplate did not show significant differences in platelet aggregation in COVID-19 patients on ventilation support compared to healthy volunteers. The aggregability samples from our study, which were collected during the pandemic from patients treated with aspirin, showed an increase in the incidence of aspiring resistance, although it was statistically insignificant, likely due to the inclusion of COVID-19-infected patients. This can, most likely, be explained by the increased synthesis of thromboxane in infected patients, which is why aspirin was initially used in the treatment of these patients to reduce thrombotic complications. However, the most recent Recovery trial did not demonstrate a reduction in mortality or the progression of respiratory failure to mechanical ventilation in patients with COVID-19 who were treated with 150 mg of aspirin daily [39].

There are few and contradictory data in the literature regarding the influence of platelet parameters on aspirin efficiency. Platelets are the target of antiplatelet drugs, so their number and characteristics play an important role in therapeutic efficacy, especially due to the different amounts of platelet cytoplasm, which is known to contain many pro-aggregating substances. In one study, the platelet count was not correlated with the ASPI test value, but only with the ADP test, in patients treated with clopidogrel and aspirin. In another, which used light transmission aggregometry (LTA), it influenced the efficacy of aspirin [40,41]. To restore clinical utility, we analyzed the platelet counts below and above the mean normal range. This way, a high platelet count predisposed patients to aspirin resistance (OR: 2.47; 95% CI: 1.23–4.97). As for the other indices, only the PCT with a value above 0.26/fL was noted as another resistance factor, as evidenced by another study [40]. However, in contrast to these studies, we did not find a significant association with MPV.

### 4.2. Treatment with P2Y12 Inhibitors

In line with current recommendations and guidelines, most patients with ACS were treated with more potent P2Y12 inhibitors: in this case, ticagrelor [4,7]. Nevertheless, in the case of certain conditions associated with an increased risk of bleeding (old age, low weight, renal dysfunction, associated drugs, or a history of bleeding), the patient was prescribed clopidogrel, the decision being made by the attending physician. This explains the difference between the characteristics of the two groups of patients, those under clopidogrel and under ticagrelor (Table 1). In our cohort, the results of the aggregation test showed, as expected, that the ADP test values of the patients being treated with clopidogrel were well above the values of patients on ticagrelor, but only two patients (5%) had HTPR (Figure 1). The incidence was very low in our study compared to the last large trial (TROPICAL-ACS), which used impedance-based aggregation, and where the incidence was about 40% [42]. In our case, this is more likely due to the selection criteria of patients receiving clopidogrel in “real life”, as mentioned above. Most patients being treated with clopidogrel were within the “therapeutic window” of antiplatelet therapy, which was also recommended by the last consensus reached [12]. On the other hand, approximately half of the patients being administered ticagrelor had decreased platelet reactivity, which is consistent with recent data from the TOPIC-VASP study [43]. It has been shown that patients with LTPR have an increased risk of bleeding, which is clearly correlated with the unfavorable prognosis and analysis of the ADEPT-DES study [44]. Thus, the patients treated with more potent antiplatelet agents are at an increased risk of bleeding throughout the treatment. This may offset their beneficial anti-ischemic effect, which is more pronounced during the acute period. Hence, the principle of the “de-escalation” of antiplatelet therapy from a more potent drug (ticagrelor; prasugrel) to clopidogrel, after the acute phase of ACS, may be applied with or without monitoring platelet function [42,43]. The first studies showed favorable results in this regard, with a reduction in the frequency of hemorrhagic events and without significantly increasing the incidence of ischemic events. This had a clearly favorable clinical benefit and low economic cost, which was confirmed by the last meta-analysis that was recently published [45,46].

In our study, due to the high percentage of LTPR patients being treated with ticagrelor, we attempted to identify the factors that influence this exaggerated antiplatelet response to aggregation tests. These factors could also be used as a clinical practice tool to individualize the antiplatelet treatment and to avoid an exaggerated response to it. Other studies have shown a direct link between age and the rate of bleeding, but it is not known to what extent this depends strictly on the degree of antiplatelet or other intricate factors (low weight, vascular fragility, other drugs, or associated co-morbidities) [47]. We have shown that old age is a factor that predisposes patients to an exaggerated response to ticagrelor, and furthermore, we have shown that being under the age of 50 can be considered a protective factor for its occurrence. The other clinical factors investigated (i.e., weight, gender, cardiovascular risk factors or diagnosis at hospital admission) did not influence the occurrence of this exaggerated response (LTPR).

On the other hand, the investigated laboratory parameters showed multiple associations in this sense. A recent study showed that platelet count influences ADP test results in patients being treated with ticagrelor [13]. Additionally, we have also demonstrated that their high number is protective against the appearance of an exaggerated antiplatelet response (LTPR). We have demonstrated that the same is true of patients with high MPV, even though the most recent studies published in the literature have not found a correlation between this index and resistance to clopidogrel or ticagrelor (HTPR) [13,48]. PCT is the index that includes the two previously mentioned ones, and its impact on aggregability has not been reported so far. It can be used as a global platelet marker to assess the risk of developing LTPR, where a high value can be considered a protective factor.

A recent study demonstrated that ADP is the main target in the development of microvascular obstruction in COVID-19 patients. Therefore, ADP receptor inhibitors could be used as therapeutic agents against thrombotic complications in these patients [49]. Nevertheless, a study published recently did not demonstrate any additional benefit of adding P2Y12 inhibitors to heparin for reducing mortality or the need for respiratory or cardiovascular support at 21 days [50]. In the second enrollment period in our study, during the COVID-19 pandemic, a more frequent use of ticagrelor, and at the same time, a decrease in the number of patients with hemorrhagic risk, was observed. This is explained by the increased platelet aggregation of patients in this period (Table 1 and Table 3). A slight increase in ADP test values was observed in patients on ticagrelor, and a significant one in those on clopidogrel during the COVID-19 pandemic. Patients who were probably infected but undiagnosed were included in the study. Ticagrelor, being more potent than clopidogrel, retained an antiplatelet effect without significant changes.

Unlike the medullary suppression effect of thienopyridines, ticagrelor has a neutral effect, according to the data published in a PLATO trial sub-study [51]. Therefore, the number of leucocytes reflects the inflammatory status of the patient, in direct connection with the type of ACS and the size of the endangered myocardium. In our study, patients with leukocytosis had a significantly lower LTPR rate, as indicated by the ADP tests, which was, possibly, also closely related to their higher inflammatory and pro-thrombotic status.

Renal dysfunction is an important indicator of ischemic complications in patients with ACS due to advanced atherosclerosis, inflammation, oxidative stress, and hyperaggregability, but also due to the underuse of antithrombotic medication and revascularization interventions [52]. On the other hand, it increases the risk of bleeding due to an overdose of drugs that are eliminated mainly in this way [44]. Ticagrelor and its active metabolite are predominantly metabolized extrarenally, so renal impairment should have little impact on them; moreover, creatinine levels may increase during treatment with ticagrelor, the mechanism of which has not yet been elucidated [53]. In our cohort, the patients treated with ticagrelor showed lower serum creatinine values less often than those being treated with clopidogrel in the context of selection bias. Still, in these patients, renal dysfunction influenced the ADP test values, showing that a significantly higher percentage of patients presented with LTPR and an implicit risk of bleeding. This analysis suggests that renal dysfunction is a predisposing factor for additional bleeding risk, but this should not limit the use of potent antiplatelet agents in these patients, who also have a much higher ischemic risk. In the PLATO study, ticagrelor was shown to be much more effective than clopidogrel in reducing ischemic events in patients with ACS, especially those with impaired renal function, without a modification in dosage and without increasing the rate of major bleeding [54].

### 4.3. Limitations of the Study

The results of the present study need to be interpreted while considering several limitations. This is an observational cohort study, and as such, it was influenced by the patient selection procedure. Thus, even though the patients were selected randomly for the study, a large percentage of patients were young, under the age of 50, which could have influenced the results. For this reason, the study should only be interpreted as a generator of hypotheses and as a clinical guidance tool. We note the small sample size and, especially, the small number of patients treated with clopidogrel, which did not allow us to carry out a statistical analysis of the factors that could have influenced its effectiveness. Some of the significant associations found may be due to confounding factors without adjusting for these using multivariable analysis.

Our study coincided with the beginning of the pandemic, which made the enrollment process difficult. Therefore, we wanted to analyze in which manner this influenced the results of the aggregometry analysis. Unfortunately, by the time the pandemic had begun, we could not screen the asymptomatic patients using molecular COVID-19 tests. That is why we could not specify in what aspect, per se, the infection could influence our results.

The lack of long-term follow-up to establish a correlation between the aggregation data and the bleeding, respectively, and ischemic events are another limitation of the study, but this should be the subject of future studies.

## 5. Conclusions

The present study provides additional evidence for the importance of using aggregometry tests due to the multiple factors influencing the action of antiplatelet therapy. Because the new antiplatelet medication is extremely effective, the patient is often exposed to a hemorrhagic risk, which must be carefully analyzed and counterbalanced with its ischemic benefits. We identified some clinical and laboratory factors that reduced the antiplatelet effect. The pandemic significantly influenced the results of the platelet aggregation tests only in patients treated with clopidogrel.

Platelet function testing remains an important tool for identifying patients that have an inadequate response to DAPT and are outside of the treatment window. Based on these results, the treatment may be optimized, and the hemorrhagic and ischemic risk significantly reduced.

## Figures and Tables

**Table 1:** General characteristics of the patients.

Parameter	Sample	Aspirin	Clopidogrel	Ticagrelor	*p* Value
*n (%)*	193 (100)	191 (98.96)	41 (21.24)	148 (76.68)	NA
Age mean ± SD	58.54 ± 13.34	58.40 ± 13.34	65.29 ± 13.84	56.74 ± 12.81	0.001
Age < 50 years *n* (%)	57 (29.53)	57 (29.84)	5 (12.19)	52 (35.13)	0.005
Men *n* (%)	138 (71.50)	137 (71.72)	28 (68.39)	109 (73.64)	0.497
Weight mean ± SD	84.14 ± 16.19	84.19 ± 16.22	83.02 ± 17.20	84.79 ± 15.91	0.541
Weight > 70 kg *n* (%)	158 (84.04)	157 (83.95)	31 (77.50)	126 (86.89)	0.142
Diagnosis					
*Myocardial infarction n (%)*	166 (86.01)	164 (85.86)	26 (63.41)	140 (94.59)	<0.001
*STEMI n (%)*	127 (65.80)	126 (65.96)	18 (43.90)	109 (73.64)	<0.001
Laboratory parameters					
*Renal dysfunction n (%)*	41 (21.57)	41 (21.80)	13 (32.50)	27 (18.49)	0.056
*Platelets > 291,000 n (%)*	43 (22.75)	43 (22.99)	8 (20.00)	34 (23.44)	0.645
*PDW median (IQR)*	12.00 (11.00–13.35)	12.00 (11.00–13.40)	12.20 (11.05–13.37)	11.90 (10.90–13.45)	0.350
*MPV median (IQR)*	10.40 (9.90–11.00)	10.40 (9.90–11.00)	10.40 (10.02–11.17)	10.40 (9.80–11.00)	0.341
*MPV > 10/fL*	128 (67.72)	127 (67.91)	30 (75.00)	95 (65.51)	0.257
*P-LCR median (IQR)*	28.40 (23.85–33.40)	28.40 (24.00–33.40)	29.15 (24.97–33.80)	28.30 (23.30–33.40)	0.342
*PCT median (IQR)*	0.26 (0.22–0.29)	0.26 (0.22–0.29)	0.24 (0.21–0.29)	0.26 (0.22–0.29)	0.231
*PCT > 0.26/fL*	86 (45.50)	85 (45.45)	13 (32.50)	70 (48.27)	0.076
*Leukocytosis n (%)*	102 (53.96)	101 (54.01)	16 (40.00)	86 (59.31)	0.030
*Anaemia n (%)*	43 (22.75)	43 (22.99)	13 (32.50)	29 (20.00)	0.095
Risk factors					
*Smoking n (%)*	70 (37.83)	69 (37.50)	5 (13.15)	64 (45.07)	<0.001
*Diabetes n (%)*	42 (21.76)	42 (21.98)	8 (19.51)	31 (20.94)	0.841
*Hypertension n (%)*	123 (63.73)	121 (63.35)	30 (73.17)	89 (60.13)	0.126
*Dyslipidemia n (%)*	131 (70.81)	129 (70.49)	25 (64.10)	102 (71.83)	0.350
*Hypercholesterolaemia n (%)*	50 (27.17)	49 (26.92)	9 (23.07)	40 (28.36)	0.511
*Hypertriglyceridaemia n (%)*	94 (51.08)	92 (50.54)	15 (38.46)	76 (53.90)	0.088
*HipoHDL n (%)*	69 (37.29)	67 (36.61)	14 (35.89)	53 (37.32)	0.870
*LDL > 70 mg/dL n (%)*	135 (73.77)	134 (74.03)	25 (64.10)	107 (76.42)	0.122
*Family history of CVDs n (%)*	41 (23.69)	41 (23.97)	5 (13.51)	36 (27.06)	0.088
*Overweight/obesity n (%)*	139 (78.08)	138 (77.97)	29 (78.37)	109 (78.98)	0.936
*ADP test median (IQR)*	NA	NA	25.00 (18.00–33.00)	19.00 (14.25–24.00)	<0.001
*LTPR n (%)*	NA	NA	12 (29.27)	70 (47.30)	0.039
*MTPR n (%)*	NA	NA	27 (65.85)	76 (51.35)	0.098
*HTPR n (%)*	NA	NA	2 (4.88)	2 (1.35)	NA
*ASPI test median (IQR)*	NA	17.00 (11.00–26.00)	24.00 (11.00–38.00)	15.50 (11.00–24.00)	0.023
*HTPR n (%)*	NA	83 (43.46)	25 (60.97)	57 (39.04)	0.012
*T2 n (%)*	85 (44.04)	85 (44.50)	14 (34.14)	70 (47.30)	0.134

The *p*-value refers to the comparison between patients treated with clopidogrel and ticagrelor. SD—standard deviation; PDW—platelet volume distribution width; MPV—mean platelet volume; P-LCR—platelet larger cell ratio; PCT—plateletcrit, STEMI—ST-Elevation Myocardial Infarction; CVDs—cardiovascular disease; NA—not applicable. Renal dysfunction: serum creatinine level higher than 1.2 mg/dL for men and higher than 0.9 mg/dL for women. IQR—interquartile range; T2—lockdown during the COVID-19 pandemic.

**Table 2:** Factors analyzed according to aspirin response.

Parameter	Total *n* = 191	Aspirin-Resistant *n* = 83 (43.46%)	Aspirin-Sensitive *n* = 108 (56.54%)	*p* Value
Age < 50 years *n* (%)	57 (29.84)	32 (56.14)	25 (43.86)	0.021
*Age mean ± SD*	*58.40* ± *13.34*	56.41 ± 13.59	59.94 ± 13.007	0.042
Men *n* (%)	137 (71.73)	66 (48.18)	71 (51.82)	0.036
Women *n* (%)	54 (28.27)	17 (31.48)	37 (68.52)	
Weight > 70 kg *n* (%)	157 (83.95)	75 (47.77)	82 (52.23)	0.013
Diagnosis				
*Myocardial infarction n (%)*	*164 (85.86)*	72 (43.90)	92 (56.10)	0.759
*STEMI n (%)*	*126 (65.96)*	60 (47.62)	66 (52.38)	0.106
Laboratory parameters				
*Renal dysfunction n (%)*	*41 (21.80)*	13 (31.71)	28 (68.29)	0.096
*Platelets > 291,000 n (%)*	43 (22.99)	26 (60.47)	17 (39.53)	0.010
*PDW median (IQR)*	*12.00 (11.00–13.40)*	12.00 (10.95–13.35)	12.00 (11.00–13.42)	0.998
*MPV median (IQR)*	*10.40 (9.90–11.00)*	10.40 (9.85–11.15)	10.40 (9.90–11.00)	0.789
*MPV > 10/fL*	*127 (67.91)*	56 (44.09)	71 (55.91)	0.754
*P-LCR median (IQR)*	28.40 (24.00–33.40)	28.70 (23.40–33.70)	28.05 (24.37–33.40)	0.808
*PCT median (IQR)*	0.26 (0.22–0.29)	0.27 (0.22–0.31)	0.25 (0.21–0.29)	0.011
*PCT > 0.26/fL*	85 (45.45)	45 (52.94)	40 (47.06)	0.015
*Anaemia n (%)*	*43 (22.99)*	20 (46.51)	23 (53.49)	0.630
*Leukocytosis n (%)*	*101 (54.01)*	54 (53.47)	47 (46.53)	0.002
Risk factors				
*Smoking n (%)*	69 (37.50)	30 (43.48)	39 (56.52)	0.908
*Diabetes n (%)*	42 (21.98)	13 (30.95)	29 (69.05)	0.064
*Hypertension n (%)*	121 (63.35)	50 (41.32)	71 (58.68)	0.434
*Dyslipidemia n (%)*	129 (70.49)	55 (42.64)	74 (57.36)	0.649
*Hypercholesterolaemia n (%)*	*49 (26.92)*	22 (44.90)	27 (55.10)	0.877
*Hypertriglyceridaemia n (%)*	*92 (50.54)*	38 (41.30)	54 (58.70)	0.466
*HipoHDL n (%)*	*67 (36.61)*	29 (43.28)	38 (56.71)	0.929
*LDL > 70 mg/dL n (%)*	*134 (74.03)*	57 (42.54)	77 (57.46)	0.447
*Family history of CVDs n (%)*	41 (23.97)	16 (39.02)	25 (60.98)	0.423
*T1 n (%)*	106 (55.50)	44 (41.51)	62 (58.49)	0.545
*T2 n (%)*	85 (44.50)	39 (45.88)	46 (54.12)	

SD—standard deviation; PDW—platelet volume distribution width; MPV—mean platelet volume; P-LCR—platelet larger cell ratio; PCT—plateletcrit; STEMI—ST-Elevation Myocardial Infarction; CVDs—cardiovascular disease. Renal dysfunction: serum creatinine level higher than 1.2 mg/dL for men and higher than 0.9 mg/dL for women. IQR—interquartile range; T1—before the COVID-19 pandemic, T2—lockdown during the COVID-19 pandemic.

**Table 3:** Investigated factors related to an elevated hemorrhagic risk * in patients receiving ticagrelor.

Parameter	Total *n* = 148	Hemorrhagic Risk *n* = 70 (47.30%)	Without Hemorrhagic Risk *n* = 78 (52.70%)	*p* Value
Age < 50 years *n* (%)	52 (35.13)	19 (36.54)	33 (63.46)	0.054
*Age mean ± SD*	56.74 ± 12.81	59.09 ± 12.15	54.64 ± 13.09	0.020
Men *n* (%)	109 (73.65)	54 (49.54)	55 (50.46)	0.361
Women *n* (%)	39 (26.35)	16 (41.03)	23 (58.97)	
Weight > 70 kg *n* (%)	126 (86.89)	60 (47.62)	66 (52.38)	0.380
Diagnosis				
*Myocardial infarction n (%)*	140 (94.59)	64 (45.71)	76 (54.28)	0.107
*STEMI n (%)*	109 (73.64)	47 (43.12)	62 (56.88)	0.089
Laboratory parameters				
*Renal dysfunction n (%)*	27 (18.49)	19 (70.37)	8 (29.63)	0.008
*Platelets > 291,000 n (%)*	34 (23.44)	10 (29.41)	24 (70.59)	0.015
*PDW median (IQR)*	11.90 (10.90–13.45)	11.80 (10.80–13.15)	11.90 (10.92–13.95)	0.516
*MPV median (IQR)*	10.40 (9.80–11.00)	10.20 (9.70–10.90)	10.50 (9.92–11.17)	0.118
*MPV > 10/fL n (%)*	95 (65.51)	39 (41.05)	56 (58.95)	0.030
*P-LCR median (IQR)*	28.30 (23.30–33.40)	26.90 (22.65–32.30)	29.05 (24.02–34.15)	0.159
*PCT median (IQR)*	0.26 (0.22–0.29)	0.24 (0.21–0.28)	0.28 (0.24–0.30)	<0.001
*PCT > 0.26/fL n (%)*	70 (48.27)	23 (32.86)	47 (67.14)	0.001
*Anaemia n (%)*	29 (20.0)	17 (58.62)	12 (41.38)	0.183
*Leukocytosis n (%)*	86 (59.31)	35 (40.70)	51 (59.30)	0.045
Risk factors				
*Smoking n (%)*	64 (45.07)	28 (43.75)	36 (56.25)	0.458
*Diabetes n (%)*	31 (20.94)	14 (45.16)	17 (54.83)	0.789
*Hypertension n (%)*	89 (60.13)	44 (49.44)	45 (50.56)	0.522
*Dyslipidemia n (%)*	102 (71.83)	44 (43.14)	58 (56.86)	0.314
*Hypercholesterolaemia n (%)*	40 (28.36)	20 (50.00)	20 (50.00)	0.489
*Hypertriglyceridaemia n (%)*	76 (53.90)	32 (42.11)	44 (57.89)	0.397
*HipoHDL n (%)*	53 (37.32)	24 (45.28)	29 (54.72)	0.928
*LDL > 70 mg/dL n (%)*	107 (76.42)	51 (47.66)	56 (52.34)	0.254
*Family history of CVDs n (%)*	36 (27.06)	13 (36.11)	23 (63.89)	0.139
*T1 n (%)*	78 (52.70)	39 (50.00)	39 (50.00)	0.487
*T2 n (%)*	70 (47.30)	31 (44.29)	39 (55.71)	

* hemorrhagic risk indicates an ADP test value less than or equal to 18 U. SD—standard deviation; PDW—platelet volume distribution width; MPV—mean platelet volume; P-LCR—platelet larger cell ratio; PCT—plateletcrit; STEMI—ST-Elevation Myocardial Infarction; CVDs—cardiovascular disease; renal dysfunction—serum creatinine level higher than 1.2 mg/dL for men and higher than 0.9 mg/dL for women. IQR—interquartile range; T1—before the COVID-19 pandemic; T2—lockdown during the COVID-19 pandemic.

**Table 4:** The influence of the pandemic on the ASPi test and ADP test values.

	Before the COVID-19 Pandemic	During the COVID-19 Pandemic	*p* Value
Aspirin			
ASPI test median (IQR)	17.00 (10.00–27.00)	16.00 (12.00–25.00)	0.745
Ticagrelor			
ADP test median (IQR)	18.50 (15.00–24.00)	20.00 (14.00–23.00)	0.881
Clopidogrel			
ADP test median (IQR)	22.00 (15.50–27.00)	31.00 (24.00–36.00)	0.042

IQR—interquartile range.

## Data Availability

Data are available from the the institutional database, upon request to the correspondent author.
